# Multiple entropy fusion predicts driver fatigue using forehead EEG

**DOI:** 10.3389/fnins.2025.1567146

**Published:** 2025-06-13

**Authors:** Renyu Yang, Ling Zhang, Renhuan Yang, Lixing Hou, Donglong Zhu, Boming Zhong

**Affiliations:** ^1^School of Informatics, Guangdong University of Finance and Economics, Guangzhou, China; ^2^Guangzhou Vocational College of Technology and Business, Guangzhou, China; ^3^College of Information Science and Technology, Jinan University, Guangzhou, China

**Keywords:** driver fatigue, forehead Electroencephalogram, multiple entropies, stacking model, signal processing

## Abstract

**Introduction:**

One promising research area in traffic safety involves the utilization of an Electroencephalogram (EEG)-based approach to assess driver fatigue in new automatic technology. However, the utilization of forehead channels for identifying fatigue has been underexplored by researchers, which limits practical application.

**Objectives:**

To assess driver fatigue using EEG signals from the forehead, we propose a novel method that combines multiple entropies with a stacking model.

**Methods:**

We collected EEG signals from 32 subjects and utilized nine entropy measures including approximate entropy, fuzzy entropy, Kolmogorov entropy, permutation entropy, sample entropy, spectral entropy, symbolic transfer entropy, wavelet log energy entropy, and wavelet packet energy entropy for feature extraction. Three fast classifiers were used to build a stacking model, including logistic regression, extreme learning machine, and light gradient boosting machine. The leave-one-out cross-validation method was used to evaluate the performance of the proposed method.

**Results:**

Our proposed method yields stronger robustness and better recognition for detecting driver fatigue, demonstrating its potential to enhance current approaches for detecting driver fatigue.

**Conclusion:**

The proposed method can provide a more effective way to detect driver fatigue.

## 1 Introduction

Driver fatigue plays a significant role in traffic accidents and fatalities, contributing to an estimated 10%–20% of all fatal road casualties (Moradi et al., 2019). With the rapid development of electronic sensors and wireless communication technology, a reliable and efficient automatic detection system has become increasingly desirable for preventing fatigue-related accidents. However, despite various proposed approaches by researchers, this remains a challenging task due to technical limitations and scenario complexity (Sikander and Anwar, 2018; McDonald et al., 2019). Existing methods have several challenges. First, contextual factors in vehicle dynamics-based technologies may affect reliability (e.g., driving skill, road geometry, vehicle characteristics) (Min et al., 2021). Second, numerous video-based detectors primarily rely on facial expressions, which can encounter difficulties under low light, harsh lighting conditions, and raise privacy concerns. Third, most psychological signal acquisition methods currently rely on tactile interaction, which might limit their practicality or user acceptance. Fourth, the recognition rate needs to be further enhanced for increased accuracy and effectiveness. Moreover, the performance of feature extractors and classification algorithms significantly impacts detection efficiency; thus, exploring new models that are both fast and stable is essential (Min et al., 2023). Lastly, enhancing credibility in complex application scenarios is also crucial for the overall reliability of driver fatigue detection systems. To improve the performance of driver fatigue detection, three aspects should be focused on increasing accuracy and reliability in future research: first, combining as multimodal measures as possible is a better way to enhance the recognition quality and make fatigue detection more reliable; second, physiological features need to be further investigated and improved, especially Electroencephalogram (EEG), which are of utmost importance to effectively monitor a driver’s fatigue status in advance (Borghini et al., 2014); and last, as the classification algorithm is the key to detection performance, new fast, stable and accurate models should be sought and adopted (Pachori et al., 2024).

Identified as one of the most crucial parameters, many feature extractors and algorithms have been employed to leverage EEG applications for driver fatigue assessment. Chuang et al. (2015) proposed and developed an EEG-based perceptual function integration network to recognize the driver’s vigilance state using spectral features, demonstrating a robust accuracy of 88%. Chai et al. (2017) used the whole EEG channels to extract autoregressive modeling features and achieved an improved accuracy of 93% through sparse-deep belief networks. In addition, Cheng et al. (2018) transformed multichannel EEG data into an image-like feature map using a fast Fourier transform and then passed maps into a convolutional neural network. In another work, proposed a novel complex framework, namely a spatiotemporal convolutional neural network, to automatically learn valid features from multichannel EEG and fulfill a classification accuracy of 97.37% (Gao et al., 2019). Ramos et al. (2022) demonstrated the potential of ensemble models in safety-critical drowsiness detection, achieving 95.2% accuracy with multi-channel EEG. Fu et al. (2016) integrated three physiological features and employed a hidden Markov model to infer driver fatigue over time. da Silveira et al. (2016) presented a methodology utilizing two new spectral power-based indicators γ/δ and (γ+ β)/(δ+ α) to assess fatigue levels based on a single EEG channel. Entropy is a measure of the degree of uncertainty in a time series and has been used extensively in the analysis of EEG data in recent years due to the non-linear and non-stationary nature of EEG signals (Acharya et al., 2015; Golui et al., 2024; Baranidharan et al., 2022). For example, Xiong et al. (2016) used the combined entropy of approximate entropy (AE) and sample entropy (SE) as features while adopting support vector machine classifier for detecting driver fatigue with the highest accuracy reaching 91.3%. Another study compared four entropy measures from multichannel EEG to study and analyze driver fatigue via four common classifiers with the highest recognition rate of 98.3% (Min et al., 2017). Hu (2017) presented a method using fuzzy entropy (FE) based on a non-frontal single channel that achieved 96.6% accuracy with a random forest classifier. However, Limited research has been conducted regarding using fewer forehead EEG-based channels to detect driver fatigue detection, presenting a significant challenge for automatic systems.

**FIGURE 1:**
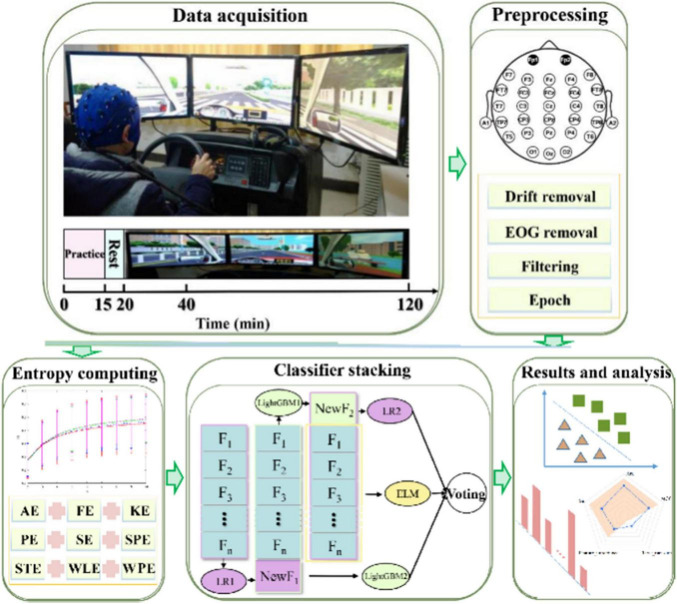
A flowchart to illustrate the operation process of assessing driving fatigue.

Although previous studies have demonstrated accurate driver fatigue detection using multichannel EEG or non-frontal electrodes (Chuang et al., 2015; Chai et al., 2017; Cheng et al., 2018; Gao et al., 2019; Fu et al., 2016; da Silveira et al., 2016; Xiong et al., 2016; Min et al., 2017; Hu, 2017), they face critical trade-offs between practicality and performance. While multichannel systems (Gao et al., 2019) achieve high accuracy, their complex setups and susceptibility to motion artifacts limit real-world adoption in driving scenarios. Recent advancements in single-channel EEG analysis highlight its viability for real-time drowsiness detection in resource-constrained environments (Balam, 2024). The prefrontal cortex is usually prioritized for channel selection due to its central role in vigilance regulation. Studies demonstrate that prefrontal theta power correlates more strongly with subjective drowsiness scores compared to central beta activity (Lin et al., 2005). Although single-channel frontal EEG improves user comfort and practicality, Relative band power (RBP) extracted via Fast Fourier Transform (FFT) methods (Li et al., 2015) underperform due to linearity assumptions. To address this, we propose a novel fusion framework combining nine complementary entropy measures with a stacking classifier for single-channel forehead EEG-based fatigue detection. This approach leverages the strengths of multiscale entropy analysis while maintaining the practicality of single-channel systems. We present a complete detection pipeline (Figure 1), including data acquisition, preprocessing, feature extraction, classification, and validation. By integrating state-of-the-art entropy measures and algorithms, we enhance the accuracy and reliability of single-channel EEG-based fatigue detection. The main contribution of this article is the fusion of multiple entropy measures and the use of a stacking classifier, which has not been explored previously in forehead EEG-based fatigue detection for drivers, and yielded a robust and enhancing recognition performance.

The organization of the paper is as follows. In see section “2 Materials and methods,” the main algorithm is derived. In see section “3 Experimental results,” experimental results are presented to evaluate the performance and superiority of the proposed method. In see section “4 Discussion,” the discussion was given. Finally, a summary of this work is given in see section “5 Conclusion.”

## 2 Materials and methods

### 2.1 Data acquisition

A total of 35 subjects (age 18–24 years old; 25 male and 10 female) were recruited to perform a simulated driving task on a static simulator. All subjects held valid C1 driver’s licenses with ≥ 1 year of driving experience (mean: 2 years; range: 1–4 years), representing a young, high-risk demographic for fatigue-related accidents. Before experiment, subjects were required to quit stimulants such as alcohol, coffee, or tea for 24 h and maintain a regular sleep schedule (≥ 7 h/night for 3 days prior). Each subject received detailed instructions on the experiment task and procedures. Written informed consent was obtained from all participants. The subjects who participated in this work were approved by the institutional research ethics committee in accordance with the declaration of Helsinki. During the experiment, subjects wore a 40-channel EEG cap (10–20 international system with two mastoid references) while seated in an acoustically shielded chamber. Although full scalp coverage was implemented, prefrontal channels (Fp1/Fp2) were prioritized for three reasons: (1) Neurophysiological relevance: Prefrontal theta activity shows stronger correlation with drowsiness scores than central beta activity (Lin et al., 2005); (2) Motion robustness: Forehead electrodes exhibit lower impedance variability (ΔZ = ± 10% vs. ± 35% in hairy regions) during head movements (Looney et al., 2012); (3) Practical feasibility: Single-channel forehead systems achieve accuracy in driving fatigue detection comparable to multi-channel setups (Chuang et al., 2018). The skin impedance was adjusted to below 5 kΩ by injecting conductive gel to start the recording process. The electrode cap included two mastoid reference channels based on the extension of the international 10–20 electrode placement standard, which had a sampling rate of 1,000 Hz (Jasper, 1958). The setup of the cap in the process of data recording is clearly introduced in Gao et al. (2019). The physical conditions and preparatory works for subjects were basically like the description in the article (Min et al., 2017). This simulator was equipped with three 24 inch color Liquid Crystal Displays and a simulation driving teaching software. The experiments were conducted on a computer equipped with an Intel Core i7-7700K CPU @ 4.20GHz, 16GB of memory, and an NVIDIA GeForce GTX 1080Ti GPU.

Similar to the experiment described in Min et al. (2017), the environment selected for this task included a highway with low traffic density, and the monotonous environment often causes fatigue. The driving time started around 14:00. Each subject had 10 min to familiarize themselves with the task scene and then freely rest for 5 min. Then, after a quick check, the subjects performed a simulated driving task for approximately 2 h. During the experiment, the subjects wearing an EEG cap performed the simulated driving task to record the EEG signal. The subjects used the Caledonian Fatigue Scale and Lee’s Subjective Fatigue Scale to report their fatigue level every 20 min (Chalder et al., 1993). Similar to previous studies, this self-reported fatigue questionnaire, including 25 questions, each with two options, can be processed quickly with a two-point scale [no (0) and yes (4)], such as “Are you drowsy?”, “Do you have difficulty concentrating?”, “Are you irritable?”, and “Are your eyes tired?” etc. In addition, video monitoring of facial expressions and a practice performance from the teaching software were also considered to conduct a comprehensive assessment for fatigue levels. Finally, each 5 min recorded EEG data in normal and fatigue state was obtained and labeled, respectively. Not all subjects exhibited signs of fatigue within the allotted window of time. During the trial, 32 out of 35 subjects were drowsy.

### 2.2 Data preprocessing

The main steps of data preprocessing were performed using Neuroscan Scan 4.3 software and MATLAB software EEGLAB toolbox after collecting raw EEG signals. As seen from Figure 1 (top right), we just selected FP1 and FP2 electrode on the forehead to preprocess. Wavelet denoising with soft thresholding was applied to suppress muscle artifacts (8–40 Hz) and eye blink components, followed by ICA-based ocular artifact removal (Jung et al., 2000). Baseline drift and electro-oculogram artifact removal was first applied to calibrate EEG data. Next, a 50 Hz notch filter and a 1–50 Hz band pass filter were performed to remove the artifacts. Electrode contact stability was monitored via amplitude variance thresholds (< 50 μV^2^). In addition, minimum-maximum normalization was applied to standardize the data to ensure that the model was not affected by amplitude differences. Then 5 min EEG signals were divided into 1 s epochs without overlap. As a result, an approximate 19,200 units for 32 subjects were produced.

### 2.3 Feature extraction

After the acquisition and preprocessing of EEG data, a suite of entropy measures was employed for feature extraction. The measures include: AE, FE, Kolmogorov entropy (KE), permutation entropy (PE), SE, spectral entropy (SPE), symbolic transfer entropy (STE), wavelet log energy entropy (WLE) and wavelet packet energy entropy (WPE). These measures were selected based on their complementary abilities to capture non-linear dynamics, spectral shifts, and network interactions in EEG signals, which are critical for detecting driver fatigue. AE and SE quantify signal regularity by measuring the probability of similar patterns recurring in EEG time series. Reduced regularity in frontal EEG during fatigue correlates with cognitive decline and attentional lapses, as demonstrated in driver fatigue studies (Min et al., 2017; Wu et al., 2012). An extension of AE/SE, FE improves robustness to EEG noise by incorporating fuzzy membership functions (Chen et al., 2009). For consistency, the embedding dimension was set to 2, and the tolerance parameter was defined as 0.7 times the standard deviation (SD) of the time series. PE evaluates complexity by analyzing ordinal patterns in EEG signals. A scale factor s = 2, embedding dimension m = 5, and a delay time τ = 4 were chosen to capture fatigue-induced shifts in long-range temporal correlations (Min et al., 2017). SPE quantifies the flatness of the EEG power spectrum, reflecting fatigue-related increases in theta/delta power and decreased beta activity (Jap et al., 2009). Unless otherwise specified, the optimal parameters for these entropy measures in this paper were determined through a grid search method. KE, on the other hand, often serves as an indicator with values that are zero for non-chaotic signals and greater than zero for chaotic signals. In this study, we employed the second-order KE for feature extraction purposes (Grassberger and Procaccia, 1983). The KE estimation is deemed both straightforward and unambiguous, as supported by a maximum-likelihood method described in Schouten et al. (1994). Through our grid search optimization, the embedding dimension m was set to 6. In accordance with the guidelines provided by Daw et al. (1995), the specific distance parameter *r_0_* was defined as Equation 1:


(1) $$\gamma_{0}=\sum_{i}\frac{\left|s_{i}-\bar{s}\right|}{N},\bar{s}=\sum_{i}\frac{s_{i}}{N}$$

where *N* denotes the number of data points, *s_i_* denotes a given N-point time series. Furthermore, to furnish energy-related information associated with EEG frequency bands, we also incorporated *WLE* and *WPE*, where the decomposition coefficients for each layer are derived through wavelet transform and wavelet packet transform. The definition of the *i-th* iteration of *WLE* can be expressed as follows Equation 2:


(2) W⁢L⁢E⁢i=∑jl⁢o⁢g⁢(ci,j2)

where *c*_*i,j*_ denotes the wavelet decomposition coefficient of the i-th leaf node in the wavelet tree. It is important to emphasize that for *WLE* construction, each individual leaf node was utilized, consequently yielding Equation 3:


(3) W⁢L⁢E=[W⁢L⁢E⁢_⁢1,W⁢L⁢E⁢_⁢2,…,W⁢L⁢E⁢_⁢i]

It can be observed that the value of *i* is the number of decomposition stages plus 1. Similarly, *WPE* is also founded upon Shannon entropy and can be defined as follows Equation 4:


(4) $$W\,P\,E=-\sum_{i}p_{i}\,{\text{log}}_{2}\,p_{i}$$

where *p_i_* is defined as the percentage of energy attributed to the wavelet packet decomposition coefficient of the *i-th* leaf node in the wavelet packet tree structure. These two wavelet entropies rests upon the selected wavelet basis function and the specified number of decomposition levels. In this study, we opted for the db3 wavelet as our basis function and set the layer count to 2. Lastly, Transfer Entropy serves as a parameter to gauge the complexity between a pair of sequences (Vicente et al., 2011). Often, a technique involving symbolic representation is employed to estimate it. For our research, we transformed EEG data into a straightforward binary sequence by marking an increment in adjacent amplitude values as “1,” while all other cases were designated as “0.” This simplification allowed us to compute STE effectively. Ultimately, we leveraged a powerful existing transfer entropy toolbox to calculate STE from the derived binary sequence (Lizier, 2014). Driver fatigue manifests through diverse EEG phenomena: temporal complexity changes (captured by AE/SE/FE/PE/KE), spectral dynamics (SPE/WLE/WPE), and network interactions (STE). The rationale for employing multi-entropy fusion lies in addressing the limitations of single-entropy approaches, which often fail to capture the heterogeneous nature of fatigue-induced EEG patterns.

### 2.4 Classification model

The entropy features extracted from each EEG epoch were input into a stacking classifier following the elimination of highly correlated attributes using Pearson’s correlation coefficient, with a stringent threshold set at 0.98. A hierarchical fusion model represents an efficacious strategy for bolstering both accuracy and robustness in regression or classification tasks. The results of different classifiers can be directly used for fusion, or the predictive result of one model can be used as the feature of another model for training and then obtain new prediction results. In this study, the stacking model was constructed as depicted in Figure 1 (central bottom panel), which integrated three fundamental classifiers: Logistic Regression (LR), Extreme Learning Machine (ELM), and light gradient boosting machine (LGBM). LR is a straightforward yet highly efficacious learning algorithm (Hosmer et al., 2013), employing the sigmoid function to map predicted values into probabilistic outcomes. ELM, on the other hand, constitutes a single-hidden layer feed-forward neural network, wherein input weights are randomly assigned and hidden layer biases only determine the output weights analytically (Huang et al., 2006). This characteristic endows it with rapid learning speed and commendable generalization capabilities. LGBM, as a gradient-boosting framework, resorts to tree-based learning algorithms, often utilizing traditional gradient boosting decision trees as its boosting component (Ke et al., 2017). It has been established as an outstanding classifier due to its numerous advantages (Ma et al., 2018). Notably, the high efficiency, inherent amenability to parallel processing, and capacity for online learning that are common to all three base classifiers render them particularly suitable for contemporary large-scale data analysis tasks. The three fundamental classifiers can be conveniently implemented through the utilization of publicly available Python libraries.

### 2.5 Evaluation method

The leave-one-out cross-validation (LOOCV) approach is widely used to assess performance and adopted to assess the detection quality for driver fatigue in this study. The well-known performance indicators, including accuracy (ACC), sensitivity (SN), specificity (SP), F1-score and area under ROC curve (AUC) were also used to provide an easier-to-understand method for assessing the classification quality (Huang and Ling, 2005). At the same time, in order to get an overview of which entropy features are most important for a model, SHapley Additive exPlanations (SHAP) values were used to show the distribution of the impacts each entropy had on the model output (Scott and Su-In, 2017).

## 3 Experimental results

The optimal performance of different entropy features based on two forehead channels using the LR benchmark classifier is shown in Table 1, which presented the performances using the four metrics mentioned above. It can be seen intuitively in Table 1 that each measure of all entropy indices was close to 0.72, implying that a single entropy for fatigue detection performed well. In addition, FE and WLE had a better overall ability to distinguish the driving states and a stronger robustness for individual differences; that the ACC and F1-scores of the former were 0.850 and 0.849, while the latter was 0.862 and 0.851, which outperformed the averages a lot. Thus, it is very suitable for using FE and WLE to study single-channel EEG data for driver fatigue detection.

**TABLE 1:** Optimal performance of different entropy features based on two forehead channels using Logistic Regression (LR) classifier.

Entropy features	ACC	SN	SP	F1-score
AE	0.698	0.688	0.709	0.693
FE	0.850	0.844	0.856	0.849
KE	0.690	0.710	0.669	0.694
PE	0.660	0.650	0.670	0.655
SE	0.697	0.687	0.707	0.692
SPE	0.680	0.648	0.711	0.666
STE	0.698	0.673	0.724	0.688
WLE	0.862	0.823	0.901	0.851
WPE	0.712	0.722	0.701	0.714
Mean ± SD	0.727 ± 0.07	0.716 ± 0.07	0.739 ± 0.08	0.723 ± 0.07

**FIGURE 2:**
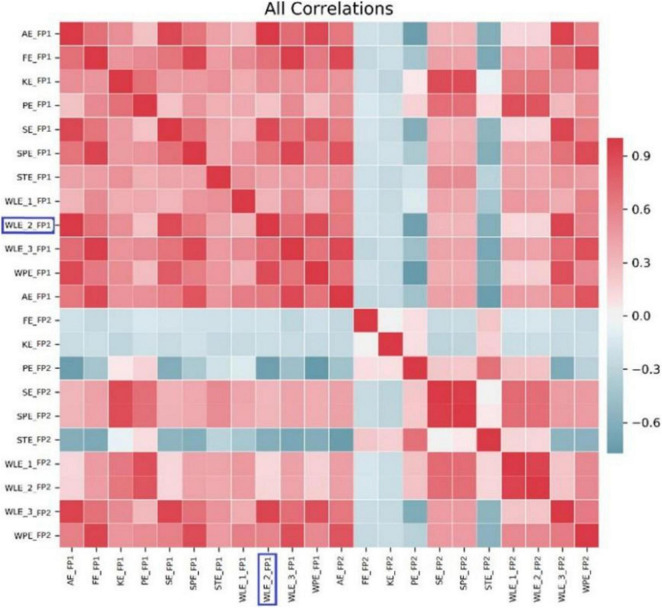
A heatmap of all coefficients above the threshold to various entropies for one subject.

In addition, for investigating multiple entropy fusion applied in predicting driver fatigue, relevant features were identified by the Pearson correlation coefficient and then removed if the coefficient was greater than a specified value before feeding to different classifiers. Figure 2 demonstrated a heatmap of all coefficients, which listed one highly correlated entropy, namely WLE_2_FP1, from all entropy pairs with a blue box for one subject. This indicated that there were common characteristics among different entropies, and the variability of entropies over time should be focalized to EEG application (Porta et al., 2001).

**FIGURE 3:**
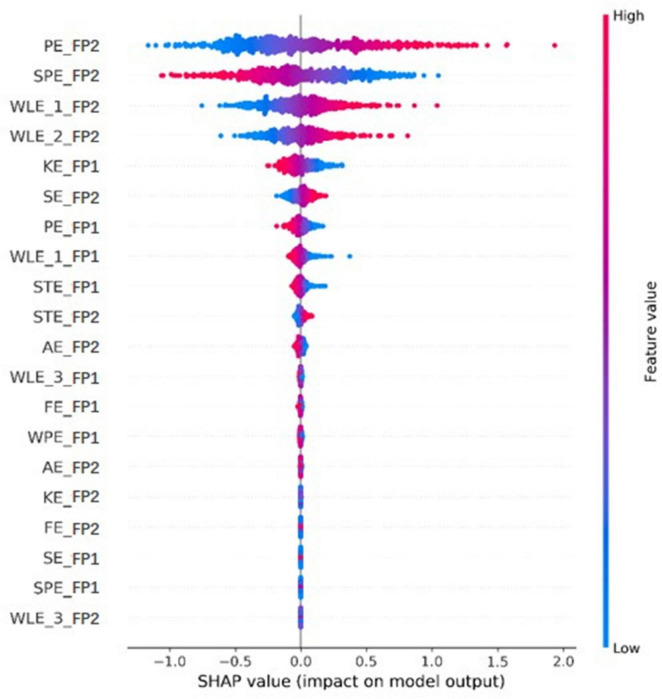
The influence of each entropy distribution on model output for the same subject in Figure 2.

As shown in Figure 3, we can also plot the SHAP values of every feature to exploit the impacts of different entropies on the model output. Corresponding to Figure 2, among these selected entropies, the size of the features represented by different colors was still distinguishable enough to approximate the normal and fatigue states. That of the last few, such as AE_FP2, KE_FP2, FE_FP2, SE_FP1, SPE_FP1, and WLE_3_FP2, was not evident, but the distribution of the features was a little different, with low feature values being the majority while FE_FP1, WPE_FP1, and AE_FP2 were the opposite. In particular, the entropy values from the top few FP2 channels had quite a bit of differentiation. For example, the high values of PE_FP2 could have a positive influence on driver fatigue detection, implying that the PE increased during the driving state change from normal to fatigue. This means the ordinal pattern could lead to an increased complexity of the EEG time series during the fatigue period. For increasing WLE_1_FP2 and WLE_2_FP2, the energy has an enhancement change to maintain the driving task and help fight fatigue from normal to fatigue (Zhang and Chen, 2012). In addition, some entropies showed a decreased phenomenon, such as SPE_FP2, KE_FP1, PE_FP1, WLE_1_FP1, and STE_FP1, in accordance with many previous studies related to entropy-reducing feedback mechanisms (Gao et al., 2018; Chen et al., 2015). However, the mechanism for analyzing the difference between FP1 and FP2 needs further study.

To rigorously evaluate the feature selection paradigm, we compared the multi-entropy fusion framework against conventional FFT-based methods. Relative FFT-RBP served as baseline inputs for both logistic regression and stacked ensemble classifiers. As empirically validated in Table 2, the entropy integration strategy demonstrated statistically superior classification accuracy (93.9% ± 5.6%) over spectral methods (71.4% ± 11.4%), with a 22.5% absolute improvement.

**TABLE 2:** Comparison of model performance with different feature extraction methods.

Feature	Classifier	ACC	Sn	Sp	F1-score	Precision	Recall
FFT	LR	0.702 ± 0.117	0.705 ± 0.117	0.700 ± 0.137	0.704 ± 0.115	0.705 ± 0.120	0.705 ± 0.117
FFT	Stacking	0.714 ± 0.114	0.718 ± 0.113	0.710 ± 0.133	0.716 ± 0.112	0.716 ± 0.118	0.718 ± 0.113
Entropy	Stacking	0.939 ± 0.056	0.939 ± 0.060	0.939 ± 0.055	0.939 ± 0.057	0.941 ± 0.054	0.936 ± 0.059

**FIGURE 4:**
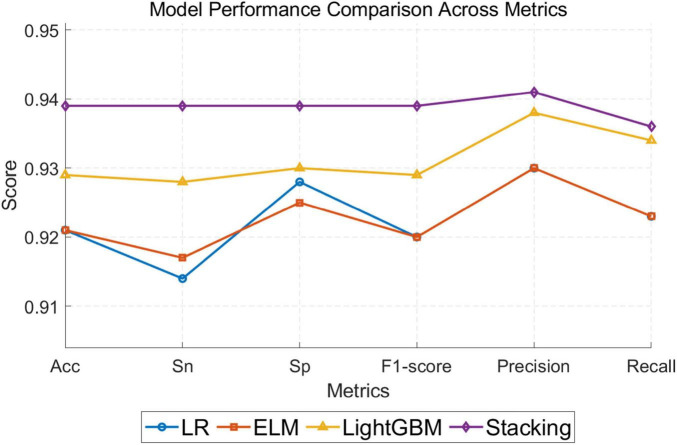
The performance metrics of the four models.

The proposed stacking model was designed to detect fatigue states for each subject, and its performances were was assessed using the LOOCV approach on EEG data sourced from the forehead channels of 32 subjects. Upon comparing the results with those obtained by the three base classifiers, Table 3 and Figure 4 presents the average recognition rates, Sensitivity, Specificity, F1-scores, Precision, Recall and their corresponding SD as determined by the stacking classifier during validation. In this table, while ELM’s performance metrics were somewhat commensurate with those of the LR benchmark classifier, it displayed a notably enhanced computational efficiency and consistently lower SD values compared to LR, thus indicating strong predictive capabilities in detecting driver fatigue with the ELM classifier. Moreover, LGBM also emerged as a highly capable detector, outperforming ELM across all six evaluation measures. However, the data in Table 3 definitively show that the stacking classifier consistently achieved the best classification performance coupled with the lowest standard deviation. Notably, when employing the two forehead channels, FP1 and FP2, the optimal average accuracy reached 93.9% with a standard deviation of 5.6%, the optimal average precision and recall rates reached 94.1% and 93.6%, respectively, which demonstrates the high reliability of the stacked model in identifying the positive class. Table 4 presents the confusion matrices of the classification results for the four classifiers. It is evident that the stacked model outperforms the other models in identifying both positive and negative classes. We conducted experiments to compare the performance of simple FFT feature extraction for logistic regression with that of the fusion of nine types of entropy for the stacked model. The results, as shown in Table 2, demonstrate that the stacked model with entropy fusion achieved an accuracy rate 23.7% higher than the logistic regression model using FFT features. This outcome underscores the superior overall performance of our proposed model in the context of classifier design.

**TABLE 3:** Results and performance using a Comparison stacking model based on two forehead channels.

Classifier	ACC	SN	SP	F1-score	Precision	Recall
LR	0.921 ± 0.079	0.914 ± 0.087	0.928 ± 0.073	0.920 ± 0.081	0.930 ± 0.072	0.923 ± 0.079
LGBM	0.929 ± 0.061	0.928 ± 0.063	0.930 ± 0.061	0.929 ± 0.614	0.938 ± 0.055	0.934 ± 0.060
ELM	0.921 ± 0.072	0.917 ± 0.076	0.925 ± 0.070	0.920 ± 0.073	0.930 ± 0.066	0.923 ± 0.070
Stacking	0.939 ± 0.056	0.939 ± 0.060	0.939 ± 0.055	0.939 ± 0.057	0.941 ± 0.054	0.936 ± 0.059

**TABLE 4:** Confusion matrix of four model.

Actual value/ Predicted value	Negative	Positive
**LR**		
Negative	8,894	793
Positive	657	8,824
**LGBM**		
Negative	8,957	623
Positive	594	8,940
**ELM**		
Negative	8,890	732
Positive	661	8,831
**STACK**		
Negative	8,980	620
Positive	571	8,943

**FIGURE 5:**
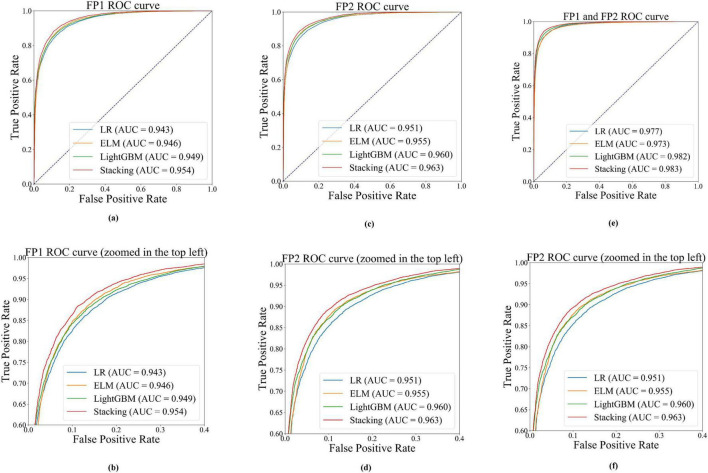
The performance of ROC and AUC for different classifiers based on the forehead channels,**(a,c,e)** represent the results obtained when utilizing FP1, FP2, and their combined signals, respectively; whereas **(b,d,f)** correspond to the zoomed-in views of the top left of sections **(a,c,e)**, respectively.

To reach a consensus on the robust stability of the obtained results, AUC was employed as the elective metric to assess the performance of driver fatigue detection, as depicted in Figure 5. As can be observed, the ROC curves corresponding to the stacking classifier consistently outperformed those of other classifiers by encompassing them. The classifier achieved AUC scores of 0.954 at FP1, 0.963 at FP2, and 0.983 when combining both channels, which are considered excellent according to AUC standards. Regarding individual classifiers, Figures 5a–d demonstrate that the performance of ELM is comparable to that of LGBM; both models surpass LR but are less effective than the stacking classifier. In Figures 5e, f, LGBM exhibits better performance compared to ELM and LR, albeit remaining inferior to the stacking model at its optimum threshold. This indicates that the stacking classifier possesses a higher level of robustness in extracting discriminative information from forehead EEG signals for detecting driver fatigue. Table 3 and Figure 5 jointly highlight that the stacking model utilizing a combination of forehead channels FP1 and FP2 achieves stronger discriminatory power compared to models using a single channel. Both metrics, F1-score and AUC, exhibit consistent performance across different classifiers when applied to data from FP1, FP2, or their combined signals. Their favorable behavior can be attributed to the versatility and robustness of entropy measures. Specifically, multiple entropy indices effectively capture the uncertainty changes in brain activity signals during the transition from normal to fatigue states while driving. Finally, the classification performance of related studies in recent years is summarized in Table 5. As observed, there is a dearth of research focusing on automatic fatigue detection using forehead electrodes (indicated by bolded accuracy values), and the reported accuracies from most articles employing frontal electrodes are not notably high. Compared with FFT-based methods, our approach achieved 11.2% higher accuracy than Li et al. (2015) and 2.6% improvement over Li et al. (2015), despite using only a single forehead channel. In comparison to previous studies, the forehead-based EEG entropy measurement method demonstrated in this study can effectively identify driver fatigue.

**TABLE 5:** Compare with other literature methods.

References	Features	Classifier	Acc (%)
Li and Chung (2015)	FFT	SVM	82.7
Mu et al. (2017)	FE	SVM	**85.0**
Li et al. (2015)	FFT	SVM-based PPM	91.3
Correa et al. (2014)	Band-based analysis	ANN	87.4
Hu (2017)	FE	RF	96.6
Wei et al. (2018)	Subband logarithmic power	SVM	**80.0**
Ogino and Mitsukura (2018)	PSD	SVM	**72.7**
Mehreen et al. (2019)	Hybrid features	SVM	92.0
This paper	Multi- entropies	A stacking model	**93.9**

The bold values indicate the accuracy of fatigue detection using forehead-mounted electrodes.

## 4 Discussion

In this study, we establish a computationally efficient stacking model that fuses multiple entropy measures to assess driver fatigue using single-channel frontal EEG signals. The framework demonstrates three key advantages for real-world deployment: First, compared with multi-channel EEG systems, frontal channel (Fp1/Fp2) selection balances neurophysiological relevance and usability by leveraging the prefrontal cortex’s established role in vigilance regulation while minimizing motion artifacts. The observed effectiveness of FE and WLE in fatigue detection using single-channel frontal EEG (ACC: 0.850–0.862) aligns with neurophysiological evidence that frontal regions correlate strongly with drowsiness scores (Lin et al., 2005). Notably, frontal electrodes are located in the prefrontal region of the brain that lacks hair, making them easier to access and more comfortable for the user, while dry electrodes here exhibit 40% lower motion-induced impedance variability than central scalp regions (Looney et al. 2012). The practicality of our forehead EEG approach could further be supported by the growing availability of low-cost, wireless EEG headsets optimized for frontal lobe recording (LaRocco et al., 2020). Second, by averaging the output of multiple classifiers with different structures, the overall risk of individual classifiers making wrong decisions can be reduced. Compared with individual classifier model, the proposed stacking model integrating LR, ELM, and LGBM, obtains more accurate and stable results. This aligns with studies demonstrating stacking’s superiority in handling physiological signal variability (Zhou, 2012), particularly for single-channel EEG applications requiring robustness to inter-subject differences (Lotte et al., 2018). Third, nine entropy measures were used for feature extraction, and the proposed model based on multi-entropy fusion can significantly improve the detection quality. Multi-entropy fusion mitigates these issues by emphasizing local signal complexity rather than global energy distribution through hybrid time-frequency and ordinal pattern analysis. For instance, WLE isolates frequency-specific non-linear dynamics via wavelet-packet decomposition, while PE filters out high-amplitude artifacts through ordinal pattern analysis. This explains our model’s 22.5% accuracy gain over FFT-PSD (Table 2) and its alignment with recent studies advocating entropy for wearable EEG (Balam, 2024).

The limitations and future work in this study can be given in the three aspects. First, although the experiment results verified the feasibility of the proposed approach with a limited number of subjects, the significant benefits of this approach need to be confirmed through further research on more subjects in real-world driving environments. Second, it is important to recognize that EEG signals represent a continuous time series with inherent temporal correlations. When accounting for local relationships within these sequences, may lead to a significant improvement in the generalization capabilities of EEG-based methods. In essence, the effective characterization and quantification of non-linear patterns in fatigue data, such as variability, sensitivity, and the evolution of temporal relationships over time, remains a substantial challenge and a key area for development in harnessing EEG technology for driver fatigue detection applications. Third, while this study primarily focuses on EEG signals for detecting driver fatigue, integrating complementary physiological signals such as electrodermal activity could potentially further improve the accuracy and reliability of fatigue detection system in future research (Veeranki et al., 2024a; Veeranki et al., 2024b). Beyond driving, our framework also could extend to neurological rehabilitation (attention tracking in ADHD therapy), supported by emerging applications in neural engineering.

## 5 Conclusion

In this paper, we introduce a novel automatic fatigue detection methodology utilizing forehead EEG signals, which is enhanced by the integration of multiple entropy measures and a fast-stacking model. The key advantage of our proposed approach lies in its ability to efficiently classify fatigue status through the utilization of entropy features derived from forehead EEG signals, using a fast-stacking classifier. From the presented results and ensuing discussions, we can draw several conclusions as follows: (1) multi-entropy fusion from forehead EEG achieves automotive-grade accuracy (94% LOOCV) comparable to commercial driver monitoring systems; (2) FE and WLE are robust for single-channel fatigue analysis, as validated by SHAP values > 0.8 for theta-band entropy features; (3) the stacking model (ELM/LGBM/LR) improves robustness, with 4.7% higher AUC than individual classifiers. Practically, this framework could be deployed in real-time driver monitoring systems to reduce fatigue-related accidents. These findings also provide a scalable solution for commercial fleets and aviation safety, advancing road safety.

## Data Availability

The original contributions presented in the study are included in the article/supplementary material, further inquiries can be directed to the corresponding author.

## References

[B1] Acharya U. R. Fujita H. Sudarshan V. K. Bhat S. Koh J. E. (2015). Application of entropies for automated diagnosis of epilepsy using EEG signals: A review. *Knowledge-based Syst.* 88 85–96. 10.1016/j.knosys.2015.08.004

[B2] Balam V. P. (2024). Systematic review of single-channel EEG-based drowsiness detection methods. *IEEE Trans. Intell. Trans. Syst.* 25 15210–15228. 10.1109/TITS.2024.3442249

[B3] Baranidharan B. Meidute-Kavaliauskiene I. Mahapatra G. S. Činčikaitė R. (2022). Assessing the sustainability of the prepandemic impact on fuzzy traveling sellers problem with a new Fermatean fuzzy scoring function. *Sustainability* 14:16560. 10.3390/su142416560

[B4] Borghini G. Astolfi L. Vecchiato G. Mattia D. Babiloni F. (2014). Measuring neurophysiological signals in aircraft pilots and car drivers for the assessment of mental workload, fatigue and drowsiness. *Neurosci. Biobehav. Rev.* 44 58–75. 10.1016/j.neubiorev.2012.10.003 23116991

[B5] Chai R. Ling S. H. San P. P. Naik G. R. Nguyen T. N. Tran Y. (2017). Improving EEG-based driver fatigue classification using sparse-deep belief networks. *Front. Neurosci.* 11:103. 10.3389/fnins.2017.00103 28326009 PMC5339284

[B6] Chalder T. Berelowitz G. Pawlikowska T. Watts L. Wessely S. Wright D. (1993). Development of a fatigue scale. *J. Psychosomatic Res.* 37 147–153. 10.1016/0022-3999(93)90081-p 8463991

[B7] Chen L. L. Zhao Y. Zhang J. Zou J. Z. (2015). Automatic detection of alertness/drowsiness from physiological signals using wavelet-based nonlinear features and machine learning. *Exp. Syst. Appl.* 42 7344–7355. 10.1016/j.eswa.2015.05.028

[B8] Chen W. T. Zhuang J. Yu W. X. Wang Z. Z. (2009). Measuring complexity using FuzzyEn, ApEn, and SampEn. *Med. Eng. Phys*. 34, 61–68. 10.1016/j.medengphy.2008.04.005 18538625

[B9] Cheng E. J. Young K. Y. Lin C. T. (2018). “Image-based EEG signal processing for driving fatigue prediction,” in *Proceedings of the 2018 international automatic control conference (CACS)*, (Piscataway, NJ: IEEE), 10.1109/cacs.2018.8606734

[B10] Chuang C. H. Cao Z. King J. T. Wu B. S. Wang Y. K. Lin C. T. (2018). Brain electrodynamic and hemodynamic signatures against fatigue during driving. *Front. Neurosci.* 12:181. 10.3389/fnins.2018.00181 29636658 PMC5881157

[B11] Chuang C. H. Huang C. S. Ko L. W. Lin C. T. (2015). An EEG-based perceptual function integration network for application to drowsy driving. *Knowledge-Based Syst.* 80 143–152. 10.1016/j.knosys.2015.01.007

[B12] Correa A. G. Orosco L. Laciar E. (2014). Automatic detection of drowsiness in EEG records based on multimodal analysis. *Med. Eng. Phys.* 36 244–249. 10.1016/j.medengphy.2013.07.011 23972332

[B13] da Silveira T. L. Kozakevicius A. J. Rodrigues C. R. (2016). Automated drowsiness detection through wavelet packet analysis of a single EEG channel. *Exp. Syst. Appl.* 55 559–565. 10.1016/j.eswa.2016.02.041

[B14] Daw C. S. Thomas J. F. Richards G. A. Narayanaswami L. L. (1995). Chaos in thermal pulse combustion. *Chaos* 5 662–670. 10.1063/1.166137 12780223

[B15] Fu R. Wang H. Zhao W. (2016). Dynamic driver fatigue detection using hidden Markov model in real driving condition. *Exp. Syst. Appl.* 63 397–411. 10.1016/j.eswa.2016.06.042

[B16] Gao Z. Li S. Cai Q. Dang W. Yang Y. Mu C. (2018). Relative wavelet entropy complex network for improving EEG-based fatigue driving classification. *IEEE Trans. Instrumentation Measurem.* 68 2491–2497. 10.1109/tim.2018.2865842

[B17] Gao Z. Wang X. Yang Y. Mu C. Cai Q. Dang W. (2019). EEG-based spatio–temporal convolutional neural network for driver fatigue evaluation. *IEEE Trans. Neural Netw. Learn. Syst.* 30 2755–2763. 10.1109/tnnls.2018.2886414 30640634

[B18] Golui S. Mahapatra B. S. Mahapatra G. S. (2024). A new correlation-based measure on Fermatean fuzzy applied on multi-criteria decision making for electric vehicle selection. *Exp. Syst. Appl.* 237:121605. 10.1016/j.eswa.2023.121605

[B19] Grassberger P. Procaccia I. (1983). Estimation of the Kolmogorov entropy from a chaotic signal. *Phys. Rev.* 28:2591. 10.1103/physreva.28.2591

[B20] Hosmer D. W. Jr. Lemeshow S. Sturdivant R. X. (2013). *Applied logistic regression.* Hoboken, NJ: John Wiley & Sons.

[B21] Hu J. (2017). Comparison of different features and classifiers for driver fatigue detection based on a single EEG channel. *Comp. Mathemat. Methods Med.* 2017:5109530. 10.1155/2017/5109530 28255330 PMC5307247

[B22] Huang G. B. Zhu Q. Y. Siew C. K. (2006). Extreme learning machine: Theory and applications. *Neurocomputing* 70 489–501. 10.1016/j.neucom.2005.12.126

[B23] Huang J. Ling C. X. (2005). Using AUC and accuracy in evaluating learning algorithms. *IEEE Trans. knowledge Data Eng.* 17 299–310. 10.1109/tkde.2005.50

[B24] Jap B. T. Lal S. Fischer P. Bekiaris E. (2009). Using EEG spectral components to assess algorithms for detecting fatigue. *ESA* 36, 2352–2359. 10.1016/j.eswa.2007.12.0

[B25] Jasper H. (1958). The 10-20 electrode system of the International Federation. *Electroencephalogr. Clin. Neuropysiol.* 10 370–375. 10.1097/00006534-195205000-00008 14941706

[B26] Jung T. P. Makeig S. Humphries C. Lee T. W. McKeown M. J. Iragui V. (2000). Removing electroencephalographic artifacts by blind source separation. *Psychophysiology* 37 163–177. 10.1111/1469-8986.3720163 10731767

[B27] Ke G. Meng Q. Finley T. Wang T. Chen W. Ma W. (2017). “Lightgbm: A highly efficient gradient boosting decision tree,” in *Proceedings of the Advances in neural information processing systems 30.* Long Beach, CA.

[B28] LaRocco J. Le M. D. Paeng D. G. (2020). A systemic review of available low-cost EEG headsets used for drowsiness detection. *Front. Neuroinform.* 15:553352. 10.3389/fninf.2020.553352 33178004 PMC7593569

[B29] Li G. Chung W. Y. (2015). A context-aware EEG headset system for early detection of driver drowsiness. *Sensors* 15 20873–20893. 10.3390/s150820873 26308002 PMC4570452

[B30] Li G. Lee B. L. Chung W. Y. (2015). Smartwatch-based wearable EEG system for driver drowsiness detection. *IEEE Sens. J.* 15 7169–7180. 10.1109/jsen.2015.2473679

[B31] Lin C. T. Wu R. C. Liang S. F. Chao W. H. Chen Y. J. Jung T. P. (2005). EEG-based drowsiness estimation for safety driving using independent component analysis. *IEEE Trans. Circuits Syst. Reg. Papers* 52 2726–2738. 10.1109/TCSI.2005.857555

[B32] Lizier J. T. (2014). JIDT: An information-theoretic toolkit for studying the dynamics of complex systems. *Front. Robot. AI* 1:11. 10.3389/frobt.2014.00011

[B33] Looney D. Kidmose P. Park C. Ungstrup M. Rank M. L. Rosenkranz K. (2012). The in-the-ear recording concept: User-centered and wearable brain monitoring. *IEEE Pulse* 3 32–42. 10.1109/MPUL.2012.2216717 23247157

[B34] Lotte F. Bougrain L. Cichocki A. Clerc M. Congedo M. Rakotomamonjy A. (2018). A review of classification algorithms for EEG-based brain–computer interfaces: A 10 year update. *J. Neural Eng.* 15:031005. 10.1088/1741-2552/aab2f2 29488902

[B35] Ma X. Sha J. Wang D. Yu Y. Yang Q. Niu X. (2018). Study on a prediction of P2P network loan default based on the machine learning LightGBM and XGboost algorithms according to different high dimensional data cleaning. *Electron. Commerce Res. Appl.* 31 24–39. 10.1016/j.elerap.2018.08.002

[B36] McDonald A. D. Alambeigi H. Engström J. Markkula G. Vogelpohl T. Dunne J. (2019). Toward computational simulations of behavior during automated driving takeovers: A review of the empirical and modeling literatures. *Hum. Fact.* 61 642–688. 10.1177/0018720819829572 30830804

[B37] Mehreen A. Anwar S. M. Haseeb M. Majid M. Ullah M. O. (2019). A hybrid scheme for drowsiness detection using wearable sensors. *IEEE Sens. J.* 19 5119–5126. 10.1109/jsen.2019.2904222

[B38] Min J. Cai M. Gou C. Xiong C. Yao X. (2023). Fusion of forehead EEG with machine vision for real-time fatigue detection in an automatic processing pipeline. *Neural Comp. Appl.* 35 8859–8872. 10.1007/s00521-022-07466-0

[B39] Min J. Wang P. Hu J. (2017). Driver fatigue detection through multiple entropy fusion analysis in an EEG-based system. *PLoS one* 12:e0188756. 10.1371/journal.pone.0188756 29220351 PMC5722287

[B40] Min J. Xiong C. Zhang Y. Cai M. (2021). Driver fatigue detection based on frontal EEG using multi-entropy measures and hybrid model. *Biomed. Signal Proc. Cont.* 69:102857. 10.1016/j.bspc.2021.102857

[B41] Moradi A. Nazari S. S. H. Rahmani K. (2019). Sleepiness and the risk of road traffic accidents: A systematic review and meta-analysis of previous studies. *Trans. Res. Part: Traffic Psychol. Behav.* 65 620–629. 10.1016/j.trf.2018.09.013

[B42] Mu Z. Hu J. Yin J. (2017). Driving fatigue detecting based on EEG signals of forehead area. *Int. J. Pattern Recogn. Art. Intell.* 31:1750011. 10.1142/s0218001417500112

[B43] Ogino M. Mitsukura Y. (2018). Portable drowsiness detection through use of a frontal single-channel electroencephalogram. *Sensors* 18:4477. 10.3390/s18124477 30567347 PMC6308812

[B44] Pachori D. Tripathy R. K. Jain T. K. (2024). Detection of atrial fibrillation from PPG sensor data using variational mode decomposition. *IEEE Sens. Lett.* 8:6001904. 10.1109/lsens.2024.3358589

[B45] Porta A. Guzzetti S. Montano N. Furlan R. Pagani M. Malliani A. (2001). Entropy, entropy rate, and pattern classification as tools to typify complexity in short heart period variability series. *IEEE Trans. Biomed. Eng.* 48 1282–1291. 10.1109/10.959324 11686627

[B46] Ramos P. M. Maior C. B. Moura M. C. Lins I. D. (2022). Automatic drowsiness detection for safety-critical operations using ensemble models and EEG signals. *Proc. Safety Environ. Protect.* 164 566–581. 10.1016/j.psep.2022.06.039

[B47] Schouten J. C. Takens F. Van Den Bleek C. M. (1994). Maximum-likelihood estimation of the entropy of an attractor. *Phys. Rev.* 49:126. 10.1103/physreve.49.126 9961199

[B48] Scott M. Su-In L. (2017). A unified approach to interpreting model predictions. *Adv. Neural Inform. Proc. Syst.* 30 4765–4774.

[B49] Sikander G. Anwar S. (2018). Driver fatigue detection systems: A review. *IEEE Trans. Intell. Trans. Syst.* 20 2339–2352. 10.1109/tits.2018.2868499

[B50] Veeranki Y. R. Diaz L. R. M. Swaminathan R. Posada-Quintero H. F. (2024a). Non-Linear signal processing methods for automatic emotion recognition using electrodermal activity. *IEEE Sens. J.* 24, 8079–8093. 10.1109/jsen.2024.3354553

[B51] Veeranki Y. R. Ganapathy N. Swaminathan R. Quintero H. F. P. (2024b). Comparison of electrodermal activity signal decomposition techniques for emotion recognition. *IEEE Access* 12 19952–19966. 10.1109/access.2024.3361832

[B52] Vicente R. Wibral M. Lindner M. Pipa G. (2011). Transfer entropy—a model-free measure of effective connectivity for the neurosciences. *J. Comp. Neurosci.* 30 45–67. 10.1007/s10827-010-0262-3 20706781 PMC3040354

[B53] Wei C. S. Wang Y. T. Lin C. T. Jung T. P. (2018). Toward drowsiness detection using non-hair-bearing EEG-based brain-computer interfaces. *IEEE Trans. Neural Syst. Rehabil. Eng.* 26 400–406. 10.1109/tnsre.2018.2790359 29432111

[B54] Wu S. D. Wu P. H. Wu C. W. Ding J. J. Wang C. C. (2012). Bearing fault diagnosis based on multiscale permutation entropy and support vector machine. *Entropy* 14 1343–1356. 10.3390/e14081343

[B55] Xiong Y. Gao J. Yang Y. Yu X. Huang W. (2016). Classifying driving fatigue based on combined entropy measure using EEG signals. *Int. J. Cont. Automat.* 9 329–338. 10.14257/ijca.2016.9.3.30

[B56] Zhang A. Chen Y. (2012). “EEG feature extraction and analysis under drowsy state based on energy and sample entropy,” in *Proceedings of the 2012 5th international conference on biomedical engineering and informatics*, (Piscataway, NJ: IEEE), 501–505. 10.1109/bmei.2012.6513081

[B57] Zhou Z.-H. (2012). *Ensemble methods: Foundations and algorithms.* Boca Raton, FL: Chapman and Hall/CRC.

